# Liver and spleen transient elastography predicts portal hypertension in patients with chronic liver disease: a prospective cohort study

**DOI:** 10.1186/s12876-015-0414-z

**Published:** 2015-12-24

**Authors:** Romanas Zykus, Laimas Jonaitis, Vitalija Petrenkienė, Andrius Pranculis, Limas Kupčinskas

**Affiliations:** Department of Gastroenterology, Lithuanian University of Health Sciences, Eivenių g. 2, Kaunas, Lithuania; Institute for Digestive Research, Lithuanian University of Health Sciences, Eivenių g. 2, Kaunas, Lithuania; Department of Radiology, Lithuanian University of Health Sciences, Eivenių g. 2, Kaunas, Lithuania

**Keywords:** Transient elastography, Portal hypertension, HVPG, Fibroscan, Non-invasive test, Spleen stiffness, Liver stiffness

## Abstract

**Background:**

To assess correlation between liver or spleen stiffness measurement by transient elastography (TE) and hepatic venous pressure gradient (HVPG) in patients with chronic liver disease as well find optimal and rule in/rule out cut-offs for prognosis of clinically significant (CSPH) and severe (SPH) portal hypertension.

**Methods:**

In this prospective study patients with different chronic liver diseases were included. TE was performed at the same day prior to HVPG measurement. HVPG was measured using catheter tip occlusion technique. Based on HVPG, patients were categorized into groups of CSPH and SPH. Cut-off values were established by applying ROC curve analysis.

**Results:**

The study included 107 consecutive patients referred for HVPG measurement or transjugular liver biopsy. Successful spleen TE was performed in 99 of the patients. Liver and spleen TE strongly correlated with HVPG, *r* = 0.75 and *r* = 0.62, respectively. Accuracy to detect CSPH was 88.7 % for liver stiffness of 17.4 kPa and 77.7 % for spleen stiffness of 47.6 kPa. Accuracy to detect SPH was 83.1 % for liver stiffness of 20.6 kPa and 77.7 % for spleen stiffness of 50.7 kPa. Liver stiffness <11.4 kPa could rule out CSPH with 55.2 % specificity and >21.9 kPa rule in CSPH with 74.4 % sensitivity. Liver stiffness <12.1 kPa could rule out SPH with 50.0 % specificity and >35 kPa rule in SPH with 58.2 % sensitivity.

**Conclusions:**

Liver and spleen stiffness correlate with HVPG and could be used to predict CSPH or SPH. Spleen elastography was not superior to liver elastography in predicting portal hypertension.

## Background

Portal hypertension is a common and important finding in patients with progressive liver disease of any etiology, leading to further complications and decreased patient survival [1, 2]. Direct measurement of portal hypertension is invasive and rarely used in clinical practice due to potential complications. Measurement of hepatic venous pressure gradient (HVPG) is established as a primary standard for evaluation of portal vein pressure, particularly in patients with hepatic (sinusoidal) portal hypertension. HVPG of less than 5 mmHg is considered to be normal, while 6–9 mmHg has been determined as subclinical portal hypertension [2]. It has shown that portal pressure can predict different outcomes in patients with portal hypertension. HVPG ≥10 mmHg was considered as clinically significant portal hypertension (CSPH) and was linked to the risk of esophageal varices formation [3], clinical decompensation [4], development of hepatocellular carcinoma (HCC) [5] or death after liver resection due to HCC [6]. Hepatic venous pressure gradient ≥12 mmHg reflected severe portal hypertension (SPH) and was found to be prognostic for acute variceal bleeding [7]. HVPG ≥16 mmHg was the predictor of poor survival in cirrhotic patients [7]. Despite the overall safety of HVPG measurement, there are some limitations related to availability of this diagnostic procedure, personal training, experience, increased health care costs and patient discomfort. Therefore, different non-invasive tests are under investigation that potentially could replace HVPG measurement. Liver stiffness could predict portal pressure by measuring physical properties of structural changes emerging in last stages of liver fibrosis; therefore, liver elastography could be potentially accurate tool for stiffness assessment. Spleen elastography has been investigated as a tool to reflect dynamic (vascular) component next to structural part of portal hypertension. Several studies have investigated diagnostic value of liver elastography; however, the role of spleen elastography has yet to be established [8–11]. The aim of our study was to assess correlation of liver and spleen stiffness measured by transient elastography (TE) with HVPG in patients with chronic liver disease. We also aimed to find optimal liver and spleen TE values as well as rule in/rule out values in predicting CSPH and SPH.

## Methods

### Study design

We performed a single center prospective study in the Gastroenterology Department at the Hospital of Lithuanian University of Health Sciences (Kaunas, Lithuania). Patients with different chronic liver diseases, who were referred for HVPG measurement or/and transjugular liver biopsy, were included in the study. As HPVG is an independent prognostic indicator in patients with chronic liver disease [3–7], patients were included irrespective of their liver biopsy status. Exclusion criteria was acute hepatitis, multiple focal liver lesions, cholestatic liver disease, biliary obstruction, failure to carry out liver transient elastography or patient’s refusal to participate in this study. Spleen elastography was performed only if liver elastography was successful. The study was approved by local Bioethics Committee of Lithuanian University of Health Sciences (Protocol No. BE-2-26). All patients have signed an informed consent form before inclusion.

### Investigations

The routine clinical (weight, height), hematological (complete blood count) and biochemical (international normalized ratio (INR), alanine aminotransferase (ALT), aspartate aminotransferase (AST), bilirubin) measurements were performed at the same day prior to HVPG measurement. Abdominal ultrasonography was performed to exclude multiple focal liver lesions.

### Non-invasive tests

Liver stiffness using FIBROSCAN® (Echosens, Paris, France) device was measured on the same day before HVPG measurement. Patients were in fasting state. Procedure was performed in accordance with manufacturer’s recommendations. Interquartile range/median <30 % and success rate >60 % was considered as good quality criteria for TE. We performed 10 successful measurements for each patient. Liver elastography was unsuccessful in 10 patients (5.9 %), who were excluded from study.

Assessment of spleen stiffness was performed by the same methodology used for liver elastography. The quality criterion (interquartile range/median, success rate and number of successful measurements) for spleen stiffness was the same as for liver stiffness. If typical elastography picture could not be found using FIBROSCAN device, exact point for spleen stiffness measurement was found using Toshiba Xario 200 ultrasound device (Toshiba Medical Systems Corporation, Japan).

### Hepatic venous pressure gradient

HVPG was measured in fasting state. None of the patients have received medications affecting portal pressure before HVPG measurement. The standard criteria for HVPG measurement was applied [12]. HPVG was measured using catheter wedge technique by experienced radiologist using judkins right 6fr catheter (Boston Scientific, USA, Marlborough). Right hepatic vein was selectively cannulated and catheter position confirmed by vein angiogram. The occluded position of the catheter was checked by absence of reflux after the injection of 2 mL of a contrast medium and appearance of sinusoidogram (Infinity R50, Drager, Germany). The mean of at least 3 readings was taken for further analysis. If the difference between the readings was greater than 1 mmHg, all the previous recordings were cancelled, and new readings were taken. Radiologist was blinded to clinical data and liver/spleen stiffness results.

### Statistical analysis

Statistical analysis was performed using SPSS 20.0. Kolmogorov – Smirnov test was used to check data normality. For descriptive statistics frequencies, means, medians and standard deviations were calculated. HVPG scores were compared with liver and spleen stiffness expressed in kPa using non-parametric Spearman correlation. Based on HVPG, patients were categorized into groups with and without CSPH or into those with and without SPH. Comparisons between patients with and without CSPH or SPH were made using Mann–Whitney Test. Areas under the receiver operating characteristic (AUROC) curve were calculated and points for best specificity and sensitivity established, positive predictive value (PPV), negative predictive value (NPV) and accuracy were calculated. P-values less than 0.05 were considered to be statistically significant.

## Results

The demographic and clinical characteristics of the patients are represented in Table 1. Patients mean age was 52.3 years, with similar gender distribution. HCV related chronic liver disease was predominant and most patients had compensated liver disease (Child Pugh grade A).

**Table 1:** Demographic and clinical characteristics of patients

	Patients (*n* = 107)
Gender, n (%)	
Females	50 (46.7)
Males	57 (53.3)
Age, years, mean (±SD)	52.3 (±11.9)
BMI, kg/m^2^, mean (±SD)	26.7 (±4.2)
Liver disease, n (%)	
Hepatitis C	68 (63.6)
Alcoholic liver disease	19 (17.8)
Cryptogenic liver disease	11 (10.3)
Other	9 (8.3)
Successful spleen elastography, n (%)	99 (92.5)
HVPG, n (%)	
≥10 mmHg	78 (72.9)
<10 mmHg	29 (27.1)
≥12 mmHg	67 (62.6)
<12 mmHg	40 (37.4)
Child Pugh score, n (%)	
A	69 (64,5)
B	32 (29,9)
C	1 (0,9)
Platelet count, /Lx10^9^, mean (±SD)	130.2 (±75.5)
ALT, IU/L, mean (±SD)	93.0 (±79.1)
AST, IU/L, mean (±SD)	105.9 (±77.6)

*SD* standard deviation, *BMI* body mass index, *HVPG* hepatic venous pressure gradient, *AST* aspartate aminotransaminase, *ALT* alanine aminotransaminase, *INR* international normalized ratio

**Fig. 1:**
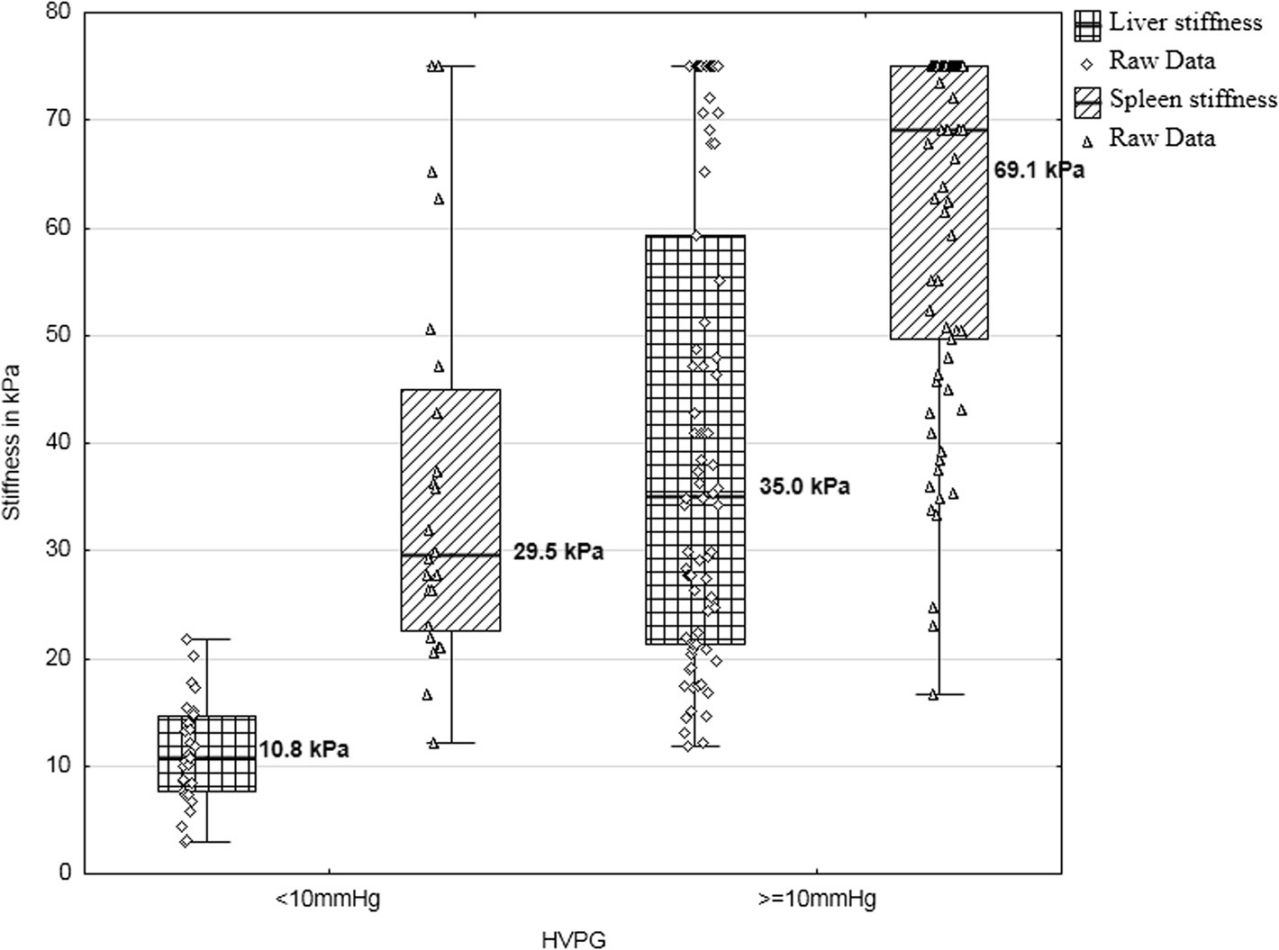
The comparison of median liver and spleen stiffness in patients with and without clinically significant portal hypertension (≥10 mmHg); *p*-value <0.01 for differences in liver stiffness; *p*-value <0.01 for differences in spleen stiffness

**Fig. 2:**
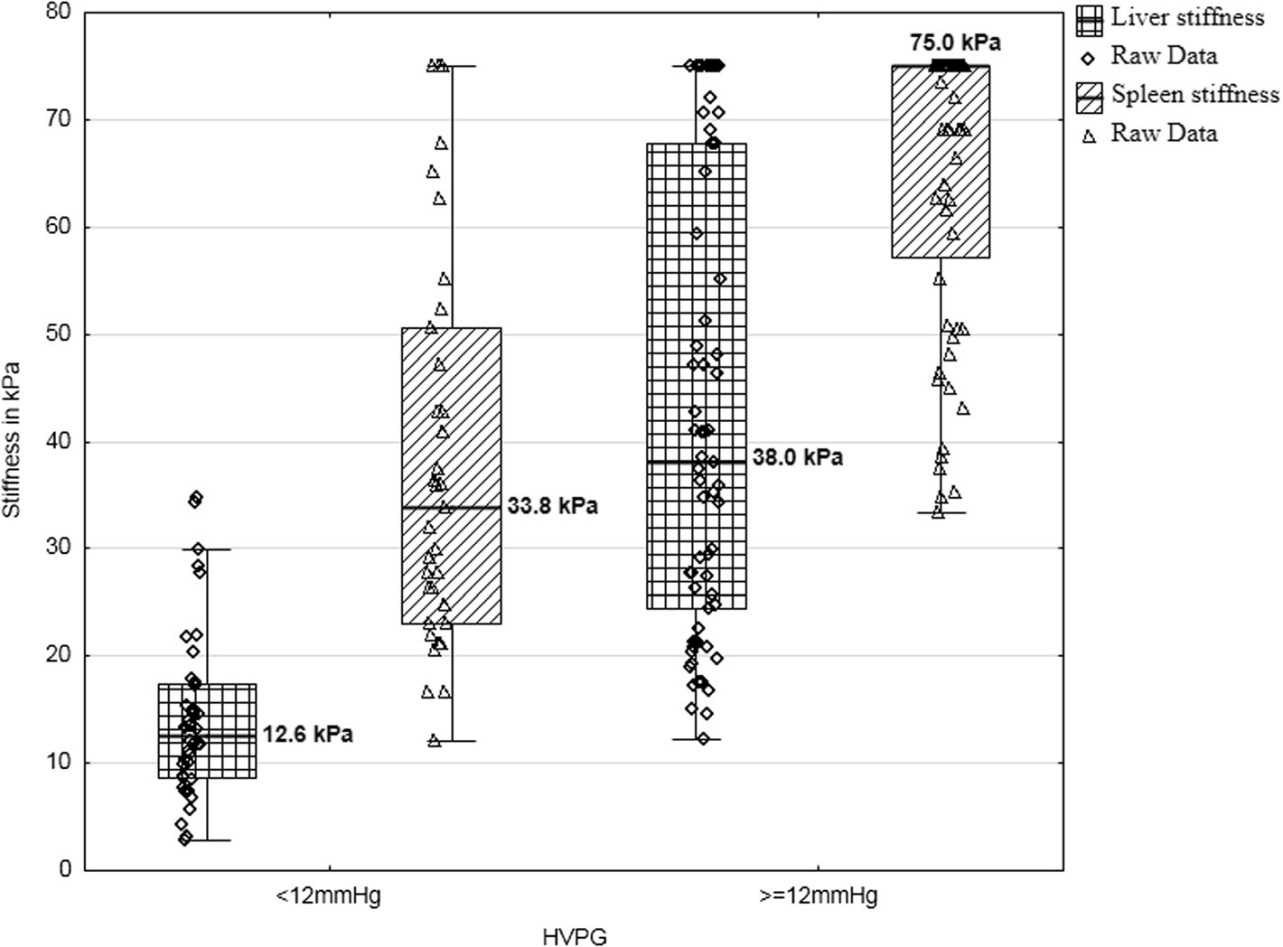
Liver and spleen stiffness comparison in patients with and without severe portal hypertension (≥12 mmHg); *p*-value <0.01 for differences in liver stiffness; *p*-value <0.01 for differences in spleen stiffness

Data of HVPG, spleen and liver elastography are represented in Table 2. Spearman correlation analysis revealed that both liver and spleen elastography correlated with hepatic venous pressure gradient. Strong correlation with HVPG was found for liver TE (r - 0.75, *p* < 0.001) and for spleen TE (r - 0.62, *p* < 0.001). Comparisons between patients with and without CSPH or SPH was made using Mann–Whitney Test. The test revealed that there are statistically significant differences in liver and spleen stiffness between patients with and without CSPH or with and without SPH (Figs. 1 and 2).

**Table 2:** Descriptives of hepatic venous pressure gradient, liver and spleen transient elastography data

	HVPG (mmHg)	Spleen TE (kPa)	Liver TE (kPa)
N	107	99	107
Std. Error of Mean	0,63	2,01	2,17
Median	14,0	62,7	25,7
Std. Deviation	6,5	20,0	22,4
Range	28	62,9	72,1
Minimum	1	12,1	2,9
Maximum	29	75,0	75,0

*HVPG* hepatic venous pressure gradient, *TE* transient elastography

**Fig. 3:**
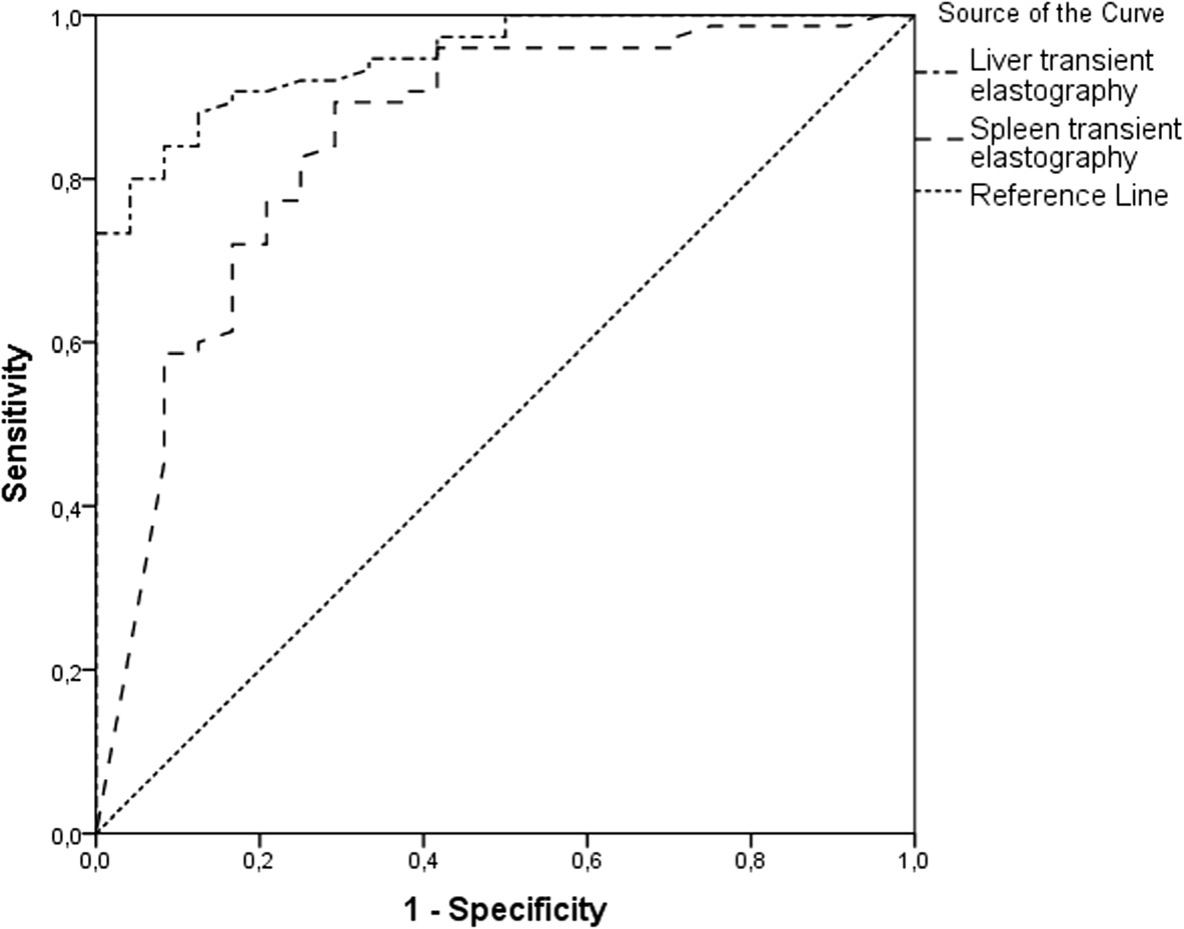
AUROC curves of liver and spleen transient elastography for clinically significant portal hypertension (≥10 mmHg)

**Fig. 4:**
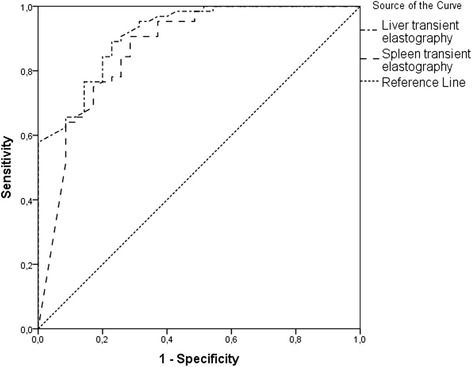
AUROC curves of liver and spleen transient elastography for severe portal hypertension (≥12 mmHg)

The AUROC curves for each HVPG category are presented in Figs. 3 and 4. AUROC curve for HVPG ≥ 10 mmHg was 0.949 (*p* < 0.001) for liver TE and 0.846 (*p* < 0.001) for spleen TE. AUROC curve for HVPG ≥ 12 mmHg was 0.915 (*p* < 0.001) for liver TE and 0.869 (*p* < 0.001) for spleen TE. AUROC curve values were higher for liver elastography than for spleen elastography and higher for CSPH than for SPH.

Liver and spleen TE cut-off values for different HVPG categories were established and specificity, sensitivity, positive predictive value, negative predictive value and accuracy were calculated (Table 3). To increase the practical utility of TE, we calculated cut-off point with 100 % specificity (rule in) or 100 % sensitivity (rule out) of liver and spleen stiffness for CSPH and SPH. The calculations revealed that there is no cut-off point with 100 % specificity for spleen stiffness both in HVPG ≥ 10 mmHg and HVPG ≥ 12 mmHg groups. Only cut-offs with 100 % sensitivity could be found, however, with little applicability. Spleen stiffness of 14.3 kPa had 4.2 % sensitivity for detecting HVPG ≥ 10 mmHg, while spleen stiffness of 32.6 kPa had 48.6 % sensitivity for HVPG ≥ 12 mmHg. In contrast to spleen TE data, analysis of liver stiffness revealed cut-off points with 100 % specificity or sensitivity in all groups (Table 4).

**Table 3:** Optimal cut-offs of liver and spleen stiffness for prediction of clinically significant (≥10 mmHg) and severe (≥12 mmHg) portal hypertension

HVPG	Method	Cut-off, kPa	Sensitivity, %	Specificity, %	PPV, %	NPV, %	Accuracy, %
≥10 mmHg	Liver TE	17.4	88.0	87.5	95.8	74.2	88.7
	Spleen TE	47.6	77.3	79.2	92.0	52.7	77.7
≥12 mmHg	Liver TE	20.6	82.8	80.0	88.8	0.75	83.1
	Spleen TE	50.7	78.1	77.1	86.2	65.8	77.7

*HVPG* hepatic venous pressure gradient, *PPV* positive predictive value, *NPV* negative predictive value

**Table 4:** Rule in and rule out cut-offs of liver stiffness for prediction of clinically significant (≥10 mmHg) and severe (≥12 mmHg) portal hypertension

HVPG	Method	Cut-off, kPa	Sensitivity, %	Specificity, %
≥10 mmHg	Liver TE	11.4	100	55.2
		21.9	74.4	100
≥12 mmHg	Liver TE	12.1	100	50.0
		35.0	58.2	100

*HVPG* hepatic venous pressure gradient, *TE* transient elastography

## Discussion

Up to date, several studies that evaluated liver elastography for prediction of clinically significant portal hypertension have been published [9–11, 13–17]. In these studies AUROC curve for prediction of CSPH varies between 0.81 and 0.94 with optimal cut-offs between 16.8 and 21.95 kPa with sensitivity and specificity of 73.7 - 89.7 % and 73.7 - 82.2 %, respectively [9, 13–15]. Both AUROC curves and optimal cut-offs observed by other authors are comparable with our data that show AUROC of 0.949 and optimal cut-off 17.4 kPa with 88 % sensitivity and 87.5 % specificity. Nevertheless, applicability of optimal cut-off points is questionable due to the risk of patient misclassification; therefore, cut-off points with 100 % sensitivity or specificity could be more useful to assess portal hypertension non-invasively, especially in HCC patients referred for surgery for evaluation of contraindications to perform liver resection. It was found that liver stiffness >29.0 kPa predicts CSPH with 71.9 % sensitivity and 100 % specificity [10]. Similar observations were made in other studies where liver stiffness of >21 kPa in HCV patients predicted CSPH with sensitivity 42 % and specificity of 100 % [16] or ≥24.2 kPa with sensitivity 52.3 % and specificity 97.1 % [18]. The rule out cut-off point was found to be <16 kPa with sensitivity 95.4 % and specificity 68.6 %. Our data revealed comparable cut-off of 21.9 kPa with sensitivity of 74.4 % and specificity of 100 %. Combining these results with the threshold of 11.4 kPa (a measure close to liver cirrhosis stage) with sensitivity of 100 % and specificity of 55.2 %, we can determine the “grey zone” between 11.4 and 21.9 kPa, and correctly classify patients outside of it. This strategy could be implemented into decision making for HCC patients, were HVPG ≥ 10 mmHg predicts higher mortality and liver dysfunction after liver resection. This group of patients could avoid unnecessary HVPG measurement if TE measurement is outside the “grey zone”.

Few studies investigated liver TE in SPH, where AUROC curve was found to be between 0.79 and 0.92 [13, 15, 19, 20]. The optimal cut-offs to predict SPH varied between 17.6 and 24.2 kPa with sensitivity and specificity of 82.9 – 94 % and 66.6 – 81 %, respectively [13, 15, 19, 20]. Our study revealed comparable results with optimal cut-off 20.6 kPa. Considering marginal cut-offs, our study revealed that rule out cut-off of 12.1 kPa had 50 % specificity, though it was almost the same as in CSPH group. Rule in cut-off point of 35.0 kPa had sensitivity of 58.2 % and it is further than CSPH cut-off.

Studies analyzing correlation of spleen stiffness measured by transient elastography with HVPG are still scarce. Colechia et al. found strong correlation between spleen stiffness and HVPG (r – 0.88) [18]. The marginal cut-off points were calculated to rule in or rule out CSPH or SPH. For CSPH cut-off 40.0 kPa had sensitivity of 98.5 % and specificity of 74.3 % and cut-off 52.8 kPa 76.9 % and 97.1 %, respectively. For SPH cut-off 41.3 kPa had sensitivity of 98.1 % and specificity of 67.4 % and cut-off 55.0 kPa - 72.2 % and 97.8 %, respectively. Our study did not show clinically useful rule in and rule out points obtained by measurement of spleen stiffness, because of wide scatter and overlap of spleen elastography in CSPH and SPH groups. In comparison, the cut-off 40 kPa in our cohort had sensitivity of 85 % and specificity of 70 % and cut-off 53.0 kPa 69.3 % and 83.3 %, respectively, for CSPH. Another study found no significant differences in spleen stiffness measured by TE between groups analyzing CSPH or SPH [8], although the study was underpowered including only 35 patients with just three patients without CSPH.

Our data show that liver and spleen elastography correlate well with HVPG, however liver stiffness has better correlation coefficient and AUROC than spleen elastography. We expected that spleen elastography might be more accurate than liver elastography for evaluation of HVPG subgroups because it reflects dynamic component of portal hypertension. Insufficient accuracy of spleen TE could be partially explained by influence of various shunts, arising during progression of portal hypertension. Taking into account other drawbacks of spleen elastography such as decreased success of spleen TE performance, missing cut-off points with 100 % specificity or sensitivity and less standardized procedure, it appears to suggest little benefit in clinical practice and no significant advantage over liver elastography. The wide variation of liver transaminases in our patients cohort could lead to higher transient elastography values and consequently to higher cut-off, as noted in different studies analyzing correlation of TE with liver fibrosis [21]. However, diagnostic accuracy of TE for cirrhosis stage is considered reliable and the influence of inflammation is less pronounced [21, 22]. Although liver biopsy was performed just in half of our patients, most of them had cirrhosis or pre-cirrhosis stage and all patients without biopsy where diagnosed as having cirrhosis based on laboratory, radiologic and clinical features. Therefore, we think that liver transaminases did not introduce significant bias into our data. To decrease the influence of inflammation to our data, we excluded all patients with acute and acute on chronic hepatitis. In our study most of the patients belonged to Child A class. Inclusion of more advanced stages of liver cirrhosis in such studies is often complicated by the presence of ascites which reflects one of the limitations of TE. The benefit of spleen stiffness measurement for evaluation of prognosis in patients with portal hypertension still has to be determined in further studies. The limitations of TE potentially could be resolved using other elastography types such as Acoustic Radiation Force Impulse imaging or Shear Wave Elastography. Further studies have to evaluate whether spleen elastography could be superior to liver elastography with these new elastography techniques for non-invasive estimation of portal hypertension [23].

## Conclusions

Our study revealed that both liver and spleen stiffness measured by transient elastography correlate with hepatic venous pressure gradient. However, spleen elastography has no advantage in comparison to standard liver elastography for prediction of clinically significant or severe portal hypertension. Liver stiffness <11.4 kPa could rule out and >21.9 kPa rule in clinically significant portal hypertension. Liver stiffness <12.1 kPa could rule out and >35 kPa rule in severe portal hypertension.

## Abbreviations

CSPH: Clinically significant portal hypertension
HVPG: Hepatic venous pressure gradient
SPH: Severe portal hypertension
HCC: Hepatocellular carcinoma
TE: Transient elastography
INR: International normalized ratio
ALT: Alanine aminotransferase
AST: Aspartate aminotransferase
AUROC: Area under the receiver operating characteristic
PPV: Positive predictive value
NPV: Negative predictive value
SD: Standard deviation
BMI: Body mass index
