# Erratum to: Liver and spleen transient elastography predicts portal hypertension in patients with chronic liver disease: a prospective cohort study

**DOI:** 10.1186/s12876-016-0467-7

**Published:** 2016-05-06

**Authors:** Romanas Zykus, Laimas Jonaitis, Vitalija Petrenkienė, Andrius Pranculis, Limas Kupčinskas

**Affiliations:** Department of Gastroenterology, Lithuanian University of Health Sciences, Eivenių g. 2, Kaunas, Lithuania; Institute for Digestive Research, Lithuanian University of Health Sciences, Eivenių g. 2, Kaunas, Lithuania

## Erratum

Following publication of the original version [1] of the article in *BMC Gastroenterology* it was brought to our attention that there is an error in Table 3. All data is presented as percentages, but the seventh column (NPV, %), third row was not converted to a %. The data is presented as 0.75, but it should be 75.

## References

[1] Zykus R (2015). Liver and spleen transient elastography predicts portal hypertension in patients with chronic liver disease: a prospective cohort study. BMC Gastroenterology.

